# Body size in early life and risk of breast cancer

**DOI:** 10.1186/s13058-017-0875-9

**Published:** 2017-07-21

**Authors:** Md. Shajedur Rahman Shawon, Mikael Eriksson, Jingmei Li

**Affiliations:** 1 Department of Medical Epidemiology and Biostatistics, Karolinska Institutet, Box 281, 171 77 Stockholm, Sweden; 2 Nuffield Department of Population Health, University of Oxford, Richard Doll Building, Old Road Campus, Oxford, OX3 7LF UK; 3 Genome Institute of Singapore, 60 Biopolis St, Genome, #02-01, Singapore, 138672 Singapore

**Keywords:** Breast cancer, Breast neoplasms, Childhood, Adolescent, Body size, Adiposity, Female, Case-control studies, Tumor characteristics

## Abstract

**Background:**

Body size in early life is inversely associated with adult breast cancer (BC) risk, but it is unclear whether the associations differ by tumor characteristics.

**Methods:**

In a pooled analysis of two Swedish population-based studies consisting of 6731 invasive BC cases and 28,705 age-matched cancer-free controls, we examined the associations between body size in early life and BC risk. Self-reported body sizes at ages 7 and 18 years were collected by a validated nine-level pictogram (aggregated into three categories: small, medium and large). Odds ratios (OR) and corresponding 95% confidence intervals (CI) were estimated from multivariable logistic regression models in case-control analyses, adjusting for study, age at diagnosis, age at menarche, number of children, hormone replacement therapy, and family history of BC. Body size change between ages 7 and 18 were also examined in relation to BC risk. Case-only analyses were performed to test whether the associations differed by tumor characteristics.

**Results:**

Medium or large body size at age 7 and 18 was associated with a statistically significant decreased BC risk compared to small body size (pooled OR (95% CI): comparing large to small, 0.78 (0.70–0.86), P_trend_ <0.001 and 0.72 (0.64–0.80), P_trend_ <0.001, respectively). The majority of the women (~85%) did not change body size categories between age 7 and 18 . Women who remained medium or large between ages 7 and 18 had significantly decreased BC risk compared to those who remained small. A reduction in body size between ages 7 and 18 was also found to be inversely associated with BC risk (0.90 (0.81–1.00)). No significant association was found between body size at age 7 and tumor characteristics. Body size at age 18 was found to be inversely associated with tumor size (P_trend_ = 0.006), but not estrogen receptor status and lymph node involvement. For all analyses, the overall inferences did not change appreciably after further adjustment for adult body mass index.

**Conclusions:**

Our data provide further support for a strong and independent inverse relationship between early life body size and BC risk. The association between body size at age 18 and tumor size could be mediated by mammographic density.

**Electronic supplementary material:**

The online version of this article (doi:10.1186/s13058-017-0875-9) contains supplementary material, which is available to authorized users.

## Background

There is a considerable amount of evidence suggesting that larger adult body size, mostly measured as body mass index (BMI), increases risk of breast cancer among postmenopausal women [1–3]; and conversely, decreases the risk among premenopausal women [1, 2]. The prevailing hypotheses for these associations include the correlation between estrogen levels and BMI and increased risk of anovulation among women with higher BMI, respectively [4–6]. There are also indications that weight change during adult life is of importance. Weight gain after menopause has been found to increase the risk of breast cancer among postmenopausal women, while weight loss after menopause has the opposite effect [3]. In addition, it has been reported that different types of weight gain in adult life (overall obesity or abdominal localization of fat) can help to explain why increased BMI is a risk marker for breast cancer in postmenopausal but not premenopausal women [7]. It is interesting to note that while high BMI and adult weight gain are associated with elevated breast cancer risk, high BMI is also associated with lower mammographic density which is a marker of decreased breast cancer risk because of higher breast fat content [8].

However, previous studies have reported that larger body size in early life appears to decrease breast cancer risk in both the premenopausal [9–14] and postmenopausal years [9, 15–17]. This relationship was also not found to be mediated by BMI in adult life [9, 10, 12, 16]. The role of body size in early life on adult breast cancer risk is of particular interest, since animal data and epidemiological research highlights the susceptibility of mammary tissue to exposures between menarche and the birth of a first child [18]. The underlying biological basis of such associations with breast cancer is not well-understood, but may be different from that of adult BMI. Since childhood adiposity is associated with earlier age at menarche [19] and earlier age at menarche is a recognized risk factor for breast cancer [20], its pronounced inverse relationship with adult breast cancer risk appears to be counterintuitive. This observed difference in risk has been postulated to be linked to the level and timing of estrogen exposure. Specifically, pre-pubertal estrogen exposure is thought to induce mammary tissue differentiation and upregulate the expression of *BRCA1* tumor suppressor gene - both of which reduce the likelihood of breast tissue becoming cancerous [21]. Other researchers have also argued that girls with larger body size in childhood experience slower adolescent growth, which decreases their risk of developing breast cancer [11]. Therefore, exploring the role of change in body size between these two periods in relation to breast cancer risk would be of importance [16].

Breast cancer is not just one disease, but a heterogeneous mixture of different tumor subtypes. Recent meta-analyses [22, 23] have established that adult body size has a differential effect on breast cancer according to estrogen and progesterone receptor (ER/PR) status, for example, increased risk of ER+/PR+ tumors. However, the current literature [9, 15–17, 24] is inconsistent on the role of body size in early life in the ER tumor subtypes. Moreover, few studies have addressed other tumor characteristics such as tumor size and/or lymph node involvement.

In this study, we used two independent Swedish breast cancer population-based studies including 6731 invasive breast cancer cases and 28,705 age-matched controls in an effort to elucidate the association between body size in early life and adult breast cancer risk, by menopausal status and tumor characteristics.

## Methods

### Study design and population

This study was based on two population-based case-control studies, namely, Karolinska mammography project for risk prediction of breast cancer (KARMA) and Linné-bröst 1 (LIBRO1). For KARMA, all women who underwent screening or clinical mammography between January 2011 and March 2013 at four participating hospitals in Sweden were invited to participate in the study. The participants were recruited from the Stockholm South General Hospital (50%), Helsingborg Hospital (27%), Skåne University Hospital, Lund (14%) and Landskrona Hospital (9%). The majority of the participants were recruited during the year 2012 (54%). During the recruitment period, a total of 210,233 women were invited to participate in the KARMA study, of whom 70,877 women (34%) joined the study. The mean ± standard deviation (SD) age at invitation and recruitment was 53.7 ± 9.9 years and 54.6 ± 10.0) years, respectively. As of October 2015, this cohort includes 3448 women with invasive breast cancer (cases), of whom 2749 (79.7%) women had prevalent cancer (i.e. the women were diagnosed before entering the study).

**Fig. 1:**
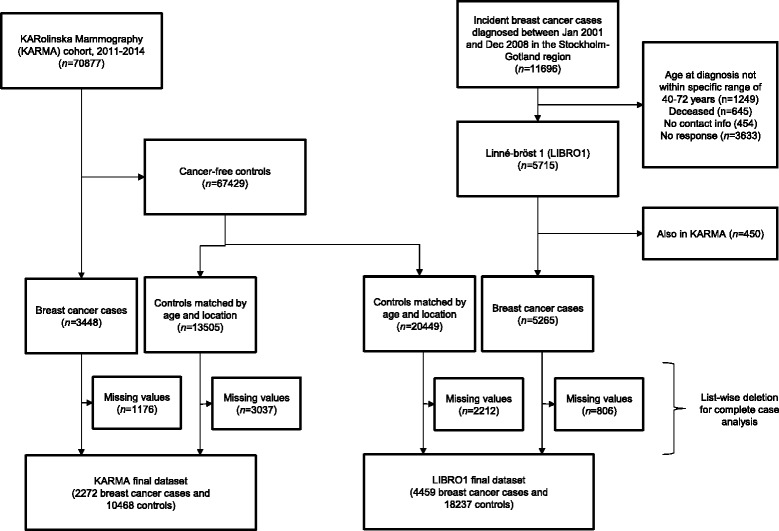
Flow of study participants in the Karolinska mammography project for risk prediction of breast cancer (*KARMA*) and Linné-bröst 1 (*LIBRO1*) case-control studies

For LIBRO1, all women diagnosed with invasive breast cancer were identified through Stockholm-Gotland Regional Breast Cancer quality register between January 2001 and December 2008. A total of 5265 women with invasive breast cancer (cases) (response rate 61%) participated in the study. There was no significant difference in the mean ages between those who participated (63.3 years) and those who did not participate (63.9 years) in the study (*P* = 0.81). Four controls were randomly selected for each KARMA/LIBRO1 case from the pool of approximately 68,000 cancer-free KARMA participants, frequency matched on age (age at diagnosis date for cases and age at questionnaire for controls, 5-year interval) and geographical location (Fig. 1). All participants filled in a detailed questionnaire on sociodemographic information, reproductive health, hormone use, lifestyle information such as tobacco and alcohol use, physical activity, diet, other diseases and related treatment. The median (interquartile range (IQR)) time differences between completion of the questionnaire and breast cancer diagnosis were 4.0 (9.0) years and 6.0 (3.0) years for KARMA and LIBRO1, respectively. The full questionnaire is available at the KARMA website (http://karmastudy.org/).

### Assessment of childhood and adolescent body sizes

**Fig. 2:**
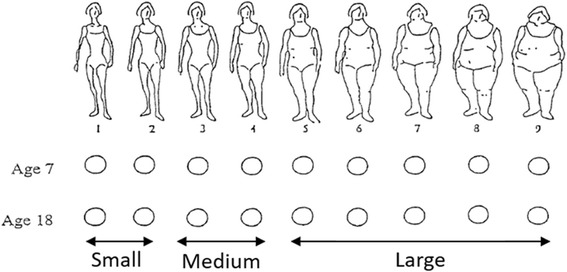
Nine-level pictogram: somatotypes for assessing body sizes at age 7 and 18 years

Information about body sizes at ages 7 and 18 years were collected in adulthood through a nine-level pictogram in the questionnaire (Fig. 2), which was validated against measured BMI in the Third Harvard Growth Study [25]. Among Swedish women, the correlation coefficients for BMI from school records and self-reported somatotypes at ages 7 and 18 were found to be 0.6 and 0.7, respectively [26]. Other studies, in different settings, also indicated that a pictogram can provide a reasonably accurate assessment of early life anthropometry [10, 16, 17, 27]. The self-reported somatotypes were aggregated into small (categories 1 and 2), medium (categories 3 and 4) and large (categories 5 to 9) prior to analysis (Fig. 2). An indicator variable on body size change (i.e. change in a major category of the pictogram) between age 7 and 18 was also created: (1) remained small (small at both age 7 and 18); (2) decreased (medium at age 7 and small at age 18 or large at age 7 and small/medium at age 18); (3) remained medium (medium at both age 7 and 18); (4) increased (small at age 7 and medium/large at age 18 or medium at age 7 and large at age 18); or (5) remained large (large both at age 7 and 18). The distributions of somatotypes at age 7 and 18 and body size category change between ages 7 and 18 are described in Additional file 1: Table S1.

### Assessment of covariates

The covariates included in this study were age at completion of the questionnaire (continuous; in years), age at menarche (continuous; in years), number of children (categorical; 0, 1, 2 or ≥3), ever use of hormone replacement therapy (HRT) (dichotomous; never/ever), family history of breast cancer in first-degree relatives including children (dichotomous; no/yes), and BMI at the time of the questionnaire response (continuous, kg/m^2^).

Menopause was defined by cessation of menstruation for 12 consecutive months, or oophorectomy. For women with missing information about menopause, an age cut off of 50 years was used to assign menopause status (<50 years premenopausal; ≥50 years postmenopausal). Women with breast cancer were considered to be postmenopausal in our analyses if they had no menstruation during the last 12 months prior to their breast cancer diagnosis. Control women were considered to be postmenopausal if they had no menstruation during the last 12 months prior to study entry.

Information on ER status, tumor size (T), lymph node involvement (N) and distant metastases (M) were retrieved from the Swedish Cancer Register for a subset of cases. The degree of missingness for information retrieved on tumor characteristics ranged between 37.8 and 43.5% in KARMA and between 21.7 and 26.9% in LIBRO1 (Additional file 1: Table S2). In our analyses, tumor size was divided into two groups (T0–T1: tumor size ≤2 cm and T2–T4: tumor size >2 cm with or without involvement of the chest wall and/or skin). Lymph node involvement was also dichotomized into “no” (N0) and “yes” (N1–N3). Presence/absence of distant metastasis was not analyzed due to the small number of such cases.

### Statistical analyses

The distributions of related demographic, reproductive health and lifestyle characteristics were described as proportions for categorical variables and mean ± SD for continuous variables in each study. The degree of missingness for each variable is provided in Additional file 1: Table S3. Analyses were adjusted for age at completion of the questionnaire and common breast cancer risk factors. These variables included age at menarche, number of children, ever use of HRT, and family history of breast cancer. Missing data were handled by list-wise deletion (complete case analysis). Figure 1 illustrates a flow diagram describing how the analytical cohorts were derived. The final analysis datasets consist of 2272 cases and 10,468 controls from KARMA, and 4459 cases and 18,237 controls from LIBRO1.

Multivariable logistic regression models were used to estimate odds ratios (ORs) and corresponding 95% confidence intervals (CI) for the associations between body size in early life and adult breast cancer risk, overall and stratified by menopausal status. In addition to the covariates described above, we also further adjusted for BMI at the time of questionnaire response in separate models to clarify whether the associations between pre-adult body size and breast cancer risk were independent of adult anthropometry. To assess trends across the small, medium and large body size categories, we assigned the median somatotype to everyone with somatotypes in that category and then included this new variable as a continuous predictor in separate logistic regression models. Heterogeneity of associations between breast cancer and body size by tumor characteristics was assessed using logistic regression analyses restricted to cases (case-only analyses) with the tumor characteristic as the outcome variable.

## Results

Table 1 describes various demographic and reproductive health characteristics of women with breast cancer (cases) and controls by study. Significantly more controls (*P* < 0.001) reported being of large body size at age 7 than women with breast cancer (KARMA: 9.5% vs. 7.0%; LIBRO1 9.6% vs. 8.4%) and age at 18 (KARMA: 9.5% vs. 7.0%; LIBRO1: 10.2% vs. 7.5%). Conversely, mean BMI at the time of questionnaire response was higher among women with breast cancer than in controls. Self-reported body size at age 7 and at age 18 was positively correlated (Pearson correlation *r* = 0.59 in KARMA; 0.60 in LIBRO1). Women with breast cancer were more likely to be postmenopausal, a larger proportion was nulliparous and more women reported family history of breast cancer compared to controls in both studies.

**Table 1:** Demographic and reproductive health characteristics of women with breast cancer (cases) and controls by study

		KARMA			LIBRO1		
Variables	Categories	Cases (*n* = 2272)	Controls (*n* = 10,468)	*P* value*	Cases (*n* = 4459)	Controls (*n* = 18237)	*P* value*
Demographics							
Age at questionnaire, years	Mean ± SD	61.3 ± 9.2	55.7 ± 9.2	<0.001	62.6 ± 9.7	57.4 ± 9.1	<0.001
Body size at age 7 years, *n* (%)	Small	1327 (58.4)	5644 (53.9)	<0.001	2727 (61.2)	9870 (54.1)	<0.001
	Medium	773 (34.0)	3824 (36.5)		1358 (30.5)	6622 (36.3)	
	Large	172 (7.6)	1000 (9.6)		374 (8.4)	1745 (9.6)	
Body size at age 18 years, *n* (%)	Small	705 (31.0)	3042 (29.1)	0.001	1482 (33.3)	5233 (28.7)	<0.001
	Medium	1407 (61.9)	6433 (61.5)		2638 (59.2)	11,135 (61.1)	
	Large	160 (7.0)	992 (9.5)		334 (7.5)	1866 (10.2)	
BMI at questionnaire, kg/m^2^	Mean ± SD	25.6 ± 4.2	25.2 ± 4.1	<0.001	25.3 ± 4.2	25.2 ± 4.2	0.071
Reproductive health factors							
Age at menarche, years	Mean ± SD	13.2 ± 1.5	13.2 ± 1.5	0.735	13.1 ± 1.5	13.1 ± 1.5	0.398
Number of children, *n* (%)	Nulliparous	407 (13.1)	1574 (11.8)	0.018	673 (15.1)	2599 (14.3)	<0.001
	1	502 (16.1)	1910 (14.3)		790 (17.7)	2746 (15.1)	
	2	1463 (46.9)	6425 (48.2)		1975 (44.3)	8425 (46.2)	
	≥3	743 (23.9)	3430 (25.7)		1021 (22.9)	4467 (24.5)	
Menopause status, *n* (%)	Postmenopause	1999 (87.9)	6733 (64.3)	<0.001	4229 (94.8)	13,143 (72.1)	<0.001
	Premenopause	273 (12.0)	3735 (35.7)		230 (5.2)	5094 (27.9)	
Ever used HRT, *n* (%)	Yes	882 (38.8)	3025 (28.9)	<0.001	2381 (53.4)	6225 (34.1)	<0.001
	No	1390 (61.2)	7443 (71.1)		2078 (46.6)	12,012 (65.9)	
Family history of breast cancer, *n* (%)	Yes	487 (21.4)	1431 (13.7)	<0.001	900 (20.2)	2426 (13.3)	<0.001
	No	1785 (78.6)	9037 (86.3)		3559 (75.4)	15,811 (86.7)	

*SD* standard deviation, *BMI* body mass index, *HRT* hormone replacement therapy. *P values were derived from chi-square tests for categorical variables and from the independent sample *t* test for continuous variables

Body size at age 7 years was inversely associated with breast cancer risk in the multivariable analysis (pooled OR comparing large to small body size: 0.78 (95% CI 0.70–0.86, P_trend_ <0.001, Table 2); with similar effects observed in both studies (KARMA OR: 0.73; 95% CI 0.61–0.87 and LIBRO1 OR: 0.80; 95% CI 0.71–0.91). The inverse association did not differ between premenopausal (OR comparing large to small: 0.72; 95% CI 0.58–0.89) and postmenopausal breast cancer (OR comparing large to small: 0.80; 95% CI 0.71–0.90) (*P* for interaction >0.05) (Table 2). Similarly, an inverse association between body size at age 18 and overall risk of breast cancer was observed in the multivariable-adjusted combined dataset (OR 0.72; 95% CI 0.64–0. 80, P_trend_ <0.001, Table 3). Similar inverse relationships were observed in analyses stratified by menopause status (Table 3). The presentation of both the age-adjusted and multivariable-adjusted results allows the reader to assess the sensitivity of age-adjusted results to the adjustment scheme. Further adjustment for adult BMI did not appreciably change the results (data not shown).

**Table 2:** Odds ratios (ORs) and corresponding 95% confidence intervals (CIs) for breast cancer risk associated with body size at age 7 years

	KARMA			LIBRO1			Combined		
Body size	Number	Age-adjusted OR (95% CI)	Multivariable OR (95% CI)^a^	Number	Age-adjusted OR (95% CI)	Multivariable OR (95% CI)^a^	Number	Age-adjusted OR (95% CI)	Multivariable OR (95% CI)^b^
Breast cancer overall									
Small	1327	1.00 (Reference)	1.00 (Reference)	2727	1.00 (Reference)	1.00 (Reference)	4054	1.00 (Reference)	1.00 (Reference)
Medium	773	0.89 (0.80–0.98)	0.88 (0.80–0.98)	1358	0.77 (0.71–0.82)	0.76 (0.71–0.82)	2131	0.81 (0.76–0.86)	0.80 (0.76–0.85)
Large	172	0.75 (0.63–0.90)	0.73 (0.61–0.87)	374	0.81 (0.72–0.92)	0.80 (0.71–0.91)	546	0.79 (0.72–0.87)	0.78 (0.70–0.86)
*P*_trend_		<0.001	<0.001		<0.001	<0.001		<0.001	<0.001
Premenopausal breast cancer									
Small	422	1.00 (Reference)	1.00 (Reference)	534	1.00 (Reference)	1.00 (Reference)	956	1.00 (Reference)	1.00 (Reference)
Medium	259	0.94 (0.77–1.14)	0.90 (0.74–1.09)	294	0.81 (0.69–0.95)	0.79 (0.67–0.92)	553	0.86 (0.76–0.97)	0.83 (0.73–0.94)
Large	51	0.63 (0.44–0.90)	0.59 (0.41–0.85)	85	0.86 (0.69–1.11)	0.80 (0.62–1.04)	136	0.77 (0.63–0.95)	0.72 (0.58–0.89)
*P*_trend_		0.015	0.004		0.039	0.020		0.003	<0.001
Postmenopausal breast cancer									
Small	905	1.00 (Reference)	1.00 (Reference)	2193	1.00 (Reference)	1.00 (Reference)	3098	1.00 (Reference)	1.00 (Reference)
Medium	514	0.89 (0.79–1.01)	0.89 (0.78–1.00)	1064	0.76 (0.70–0.83)	0.76 (0.70–0.83)	1578	0.80 (0.75–0.86)	0.80 (0.75–0.86)
Large	121	0.80 (0.65–0.99)	0.79 (0.64–0.98)	289	0.80 (0.69–0.92)	0.80 (0.70–0.93)	410	0.80 (0.71–0.90)	0.80 (0.71–0.90)
*P*_trend_		0.018	0.014		<0.001	<0.001		<0.001	<0.001

Number is the number of breast cancer cases

^a^Adjusted for age, age at menarche, family history of breast cancer, number of children and ever use of hormone replacement therapy

^b^Adjusted for study, age, age at menarche, family history of breast cancer, number of children and ever use of hormone replacement therapy

**Table 3:** Odds ratios (ORs) and corresponding 95% confidence intervals (CIs) for breast cancer risk associated with body size at age 18 years

	KARMA			LIBRO1			Combined		
Body size	Number	Age-adjusted OR (95% CI)	Multivariable OR (95% CI)^a^	Number	Age-adjusted OR (95% CI)	Multivariable OR (95% CI)^a^	Number	Age-adjusted OR (95% CI)	Multivariable OR (95% CI)^b^
Breast cancer overall									
Small	705	1.00 (Reference)	1.00 (Reference)	1482	1.00 (Reference)	1.00 (Reference)	2187	1.00 (Reference)	1.00 (Reference)
Medium	1407	0.97 (0.87–1.07)	0.95 (0.86–1.06)	2638	0.85 (0.79–0.91)	0.85 (0.79–0.91)	4045	0.89 (0.84–0.94)	0.88 (0.83–0.94)
Large	160	0.79 (0.66–0.96)	0.78 (0.64–0.94)	334	0.68 (0.59–0.78)	0.69 (0.60–0.79)	494	0.72 (0.65–0.80)	0.72 (0.64–0.80)
*P*_trend_		0.005	0.013		<0.001	<0.001		<0.001	<0.001
Premenopausal breast cancer									
Small	214	1.00 (Reference)	1.00 (Reference)	284	1.00 (Reference)	1.00 (Reference)	498	1.00 (Reference)	1.00 (Reference)
Medium	466	1.06 (0.87–1.31)	1.03 (0.84–1.28)	545	0.82 (0.70–0.97)	0.80 (0.68–0.94)	1011	0.91 (0.80–1.03)	0.88 (0.78–1.00)
Large	52	0.70 (0.49–1.00)	0.66 (0.46–0.95)	84	0.66 (0.50–0.86)	0.62 (0.47–0.81)	136	0.67 (0.54–0.83)	0.63 (0.51–0.78)
*P*_trend_		0.190	0.043		<0.001	<0.001		<0.001	<0.001
Postmenopausal breast cancer									
Small	491	1.00 (Reference)	1.00 (Reference)	1198	1.00 (Reference)	1.00 (Reference)	1689	1.00 (Reference)	1.00 (Reference)
Medium	941	0.94 (0.83–1.07)	0.93 (0.82–1.06)	2093	0.83 (0.76–0.91)	0.84 (0.77–0.91)	3034	0.87 (0.81–0.93)	0.87 (0.81–0.93)
Large	108	0.87 (0.69–1.09)	0.86 (0.68–1.09)	250	0.67 (0.57–0.78)	0.69 (0.59–0.81)	358	0.72 (0.63–0.82)	0.74 (0.64–0.84)
*P*_trend_		0.065	0.142		<0.001	<0.001		<0.001	<0.001

Number is the number of breast cancer cases

^a^Adjusted for age, age at menarche, family history of breast cancer, number of children and ever use of hormone replacement therapy

^b^Adjusted for study, age, age at menarche, family history of breast cancer, number of children and ever use of hormone replacement therapy

The majority of the women (~85%) did not change body size categories between age 7 and 18 years (see Additional file 1: Table S1). Approximately 12% of the women had a decrease in body size category, while less than 2% experienced an increase. The strongest inverse relationship with adult breast cancer was in women who remained large at both time points when compared to women who remained small (pooled multivariable OR 0.67; 95% CI 0.57–0.79, Table 4). Significant inverse associations were also observed in women who remained medium compared to women who remained small (multivariable OR 0.79; 95% CI 0.73–0.85). A reduction in body size category was also found to be inversely associated with breast cancer risk (multivariable OR 0.90; 95% CI 0.81–1.00). Similarly, further adjustment for adult BMI did not appreciably change the results (data not shown).

**Table 4:** Odds ratios (ORs) and corresponding 95% confidence intervals (CI) for the association between body size change from childhood to adolescence and overall risk of breast cancer

	KARMA			LIBRO1			Combined		
Body size change	*n*	Age-adjusted OR (95% CI)	Multivariable OR (95% CI)^a^	*n*	Age-adjusted OR (95% CI)	Multivariable OR (95% CI)^a^	*n*	Age-adjusted OR (95% CI)	Multivariable OR (95% CI)^b^
Remained small	615	1.00 (Reference)	1.00 (Reference)	1320	1.00 (Reference)	1.00 (Reference)	1935	1.00 (Reference)	1.00 (Reference)
Decrease	181	0.92 (0.82–1.05)	0.89 (0.73–1.07)	382	0.92 (0.81–1.05)	0.91 (0.79–1.04)	563	0.92 (0.83–1.02)	0.90 (0.81–1.00)
Remained medium	626	0.93 (0.82–1.05)	0.91 (0.80–1.03)	1067	0.73 (0.67–0.80)	0.73 (0.66–0.80)	1693	0.79 (0.74–0.85)	0.79 (0.73–0.85)
Increase	784	1.06 (0.94–1.19)	1.04 (0.92–1.17)	1555	0.91 (0.83–0.99)	0.91 (0.84–0.99)	2339	0.95 (0.89–1.02)	0.95 (0.89–1.02)
Remained large	66	0.78 (0.59–1.03)	0.75 (0.56–0.99)	130	0.63 (0.51–0.77)	0.63 (0.52–0.77)	196	0.67 (0.57–0.79)	0.67 (0.57–0.79)

*n* number of breast cancer cases

^a^Adjusted for age, age at menarche, family history of breast cancer, number of children and ever use of hormone replacement therapy

^b^Adjusted for study, age, age at menarche, family history of breast cancer, number of children and ever use of hormone replacement therapy

Body size at age 7 years was not found to influence ER status, tumor size or lymph node status (Table 5). Similarly, body size at age 18 was not associated with the aforementioned tumor characteristics except for tumor size (Table 6). Larger body size at age 18 was significantly associated with smaller tumors.

**Table 5:** Odds ratios (ORs) and corresponding 95% confidence intervals (CI) for the associations between body size at age 7 years and tumor characteristics of breast cancer (case-only analysis)

	KARMA			LIBRO1			Combined		
	Number		Multivariable OR (95% CI)^a^	Number		Multivariable OR (95% CI)^a^	Number		Multivariable OR (95% CI)^b^
ER status									
	ER-negative	ER-positive		ER-negative	ER-positive		ER-negative	ER-positive	
Body size, age 7									
Small	107	848	1.00 (Reference)	317	1958	1.00 (Reference)	424	2806	1.00 (Reference)
Medium	81	514	0.91 (0.63–1.30)	184	966	0.87 (0.71–1.07)	265	1480	0.89 (0.74–1.06)
Large	20	107	0.56 (0.31–0.99)	43	270	1.02 (0.71–1.46)	63	377	0.88 (0.65–1.19)
*P*_trend_			0.062			0.717			0.234
Tumor size									
	T0–T1	T2–T4		T0–T1	T2–T4		T0–T1	T2–T4	
Body size, age 7									
Small	729	325	1.00 (Reference)	1482	656	1.00 (Reference)	2211	981	1.00 (Reference)
Medium	471	176	0.86 (0.66–1.10)	757	327	0.95 (0.80–1.12)	1228	503	0.92 (0.80–1.05)
Large	101	37	0.70 (0.43–1.15)	211	100	0.98 (0.75–1.29)	312	137	0.90 (0.71–1.15)
*P*_trend_			0.098			0.762			0.255
Lymph node involvement									
	No	Yes		No	Yes		No	Yes	
Body size, age 7									
Small	843	195	1.00 (Reference)	1889	237	1.00 (Reference)	2732	432	1.00 (Reference)
Medium	542	108	0.93 (0.69–1.25)	943	134	1.14 (0.90–1.44)	1485	242	1.08 (0.90–1.29)
Large	110	27	1.24 (0.74–2.09)	277	33	0.99 (0.66–1.47)	387	60	1.06 (0.77–1.44)
*P*_trend_			0.611			0.769			0.572

Number is the number of cases. ER estrogen receptor, *T* tumor size

^a^Adjusted for age, age at menarche, number of children, ever use of hormone replacement therapy and family history of breast cancer

^b^Adjusted for age, age at menarche, number of children, ever use of hormone replacement therapy, family history of breast cancer and study

**Table 6:** Odds ratios (ORs) and corresponding 95% confidence intervals (CI) for the associations between body size at age 18 years and tumor characteristics of breast cancer (case-only analysis)

	KARMA			LIBRO1			Combined		
	Number		Multivariable OR (95% CI)^a^	Number		Multivariable OR (95% CI)^a^	Number		Multivariable OR (95% CI)^b^
ER status									
	ER-negative	ER-positive		ER-negative	ER-positive		ER-negative	ER-positive	
Body size, age 18									
Small	58	463	1.00 (Reference)	164	994	1.00 (Reference)	222	1457	1.00 (Reference)
Medium	142	927	0.85 (0.59–1.24)	311	1733	0.91 (0.74–1.12)	453	2660	0.90 (0.76–1.08)
Large	11	119	1.05 (0.50–2.19)	37	222	1.00 (0.68–1.47)	48	341	1.01 (0.72–1.43)
*P*_trend_			0.880			0.783			0.791
Tumor size									
	T0–T1	T2–T4		T0–T1	T2–T4		T0–T1	T2–T4	
Body size, age 18									
Small	368	190	1.00 (Reference)	739	329	1.00 (Reference)	1107	519	1.00 (Reference)
Medium	867	321	0.75 (0.58–0.96)	1365	587	0.97 (0.82–1.14)	2232	908	0.90 (0.78–1.03)
Large	99	38	0.54 (0.32–0.92)	174	72	0.87 (0.64–1.18)	273	110	0.77 (0.59–1.00)
*P*_trend_			0.008			0.379			0.030
Lymph node involvement									
	No	Yes		No	Yes		No	Yes	
Body size, age 18									
Small	447	108	1.00 (Reference)	955	107	1.00 (Reference)	1402	215	1.00 (Reference)
Medium	980	196	0.83 (0.62–1.12)	1700	239	1.28 (1.00–1.63)	1219	435	1.10 (0.91–1.33)
Large	108	29	1.21 (0.70–2.07)	213	32	1.30 (0.85–1.99)	321	61	1.26 (0.91–1.76)
*P*_trend_			0.812			0.122			0.140

Number is the number of cases. ER estrogen receptor, *T* tumor size

^a^Adjusted for age, age at menarche, number of children, ever use of hormone replacement therapy and family history of breast cancer

^b^Adjusted for age, age at menarche, number of children, ever use of hormone replacement therapy, family history of breast cancer and study

## Discussion

In summary, larger body sizes at age 7 and 18 years were both associated with reduced risk of both premenopausal and postmenopausal breast cancer. Larger body size at age 18 was significantly associated with smaller tumors.

Our findings on the relationship between body size in early life and breast cancer risk agree with most of the epidemiological studies, both in direction and magnitude. Results from worldwide, large, prospective studies [9–11, 14, 16] support evidence that various measures of body fatness in early life (i.e. as determined by the nine-level pictogram, perceived relative body fatness and BMI) are inversely associated with adult breast cancer risk. Retrospective case-control studies also reported similar protective roles of body fatness at young ages [12, 13, 17, 26, 28–30]. A record-linkage study involving 117,415 Danish women reported low BMI was an independent risk factor for breast cancer [31]. In the Swedish context, we have shown previously in an independent case-control study (CAHRES) that large body size at age 7 was associated with 27% reduction in the risk of postmenopausal breast cancer when compared to the small category [17]. After adjusting for adult BMI in this study, the associations remained significant and did not appreciably differ, suggesting larger body size in early life may confer an independent long-lasting protection against breast carcinogenesis.

Larger body size during the pre-pubertal period can influence sexual maturation and pubertal growth, which mediate the inverse association with breast cancer risk in adulthood. Reports from the French E3N cohort suggested a unique effect of body fatness at menarche on breast carcinogenesis, irrespective of body fatness at other ages [16]. Similarly, our data suggest that larger body size at age 7 is significantly associated with earlier age at menarche (data not shown). Since earlier age at menarche is considered to increase the cumulative exposure to estrogen, childhood adiposity should intuitively be associated with increased breast cancer risk. However, early puberty (i.e. age at menarche) also appears to result in shorter final height (which is also a risk factor for breast cancer) in observational studies [32].

While childhood body size is well-known for its inverse relationship with adult breast cancer risk, there is less literature available on body size change in early life. Our data suggested that those women who had reduction in their body size between age 7 and 18 years had lower odds of breast cancer compared to those who remained small. An increase in body size from childhood to adolescence did not result in any significant reduction in breast cancer risk. However, others have found that a large body size at menarche reduces postmenopausal risk irrespective of body size at other time points (before or after menarche) [16]. Berkey et al. also showed that although childhood body fatness was associated with lower adult breast cancer risk, increasing body fatness between ages 10 and 20 years was not protective against either premenopausal or postmenopausal breast cancer [11]. It should however be noted that the proportion of women changing body size categories in our study is small compared to those who did not change. While it is conceivable that the majority of the population do not change major categories of body size across life, recall bias resulting from the collection of somatotype information many years later may also lead to individuals reporting a more consistent body size pattern and less change over time. Further studies will be required to confirm our results.

Estrogen, depending on the timing of exposure, is thought to have a dual impact on breast carcinogenesis [21]. Though cumulative exposure to estrogen in the post-pubertal period is considered a well-established risk factor for breast cancer [4], exposure to estrogen in the pre-pubertal years is hypothesized to confer protection against breast cancer. Large pre-pubertal girls are thought to have higher level of bioavailable estradiol possibly due to both increased production of estrogen by aromatase in adipose tissue and lower plasma concentration of sex-hormone binding globulin [11, 21]. Several animal studies [33, 34] revealed lower incidence of mammary tumors in rats after pre-pubertal or pubertal exposure to estrogen. Furthermore, several studies have postulated that pre-pubertal estrogenic exposure may reduce adult breast cancer risk by inducing persistent upregulation of a tumor suppressor gene (*BRCA1*) in the mammary gland [35, 36].

However, if we consider the ovaries as a major source of estrogen production after puberty, then the estrogen-induced causal pathway will play a less prominent role in the association between body size at age 18 and breast cancer. An alternative explanation for the inverse relationship between adolescent body size and breast cancer risk might be due to increased serum insulin and androgen levels [37], which can lead to anovulatory menstrual cycles and reduced exposure to sex hormones [38, 39]. Recent evidence also suggests that adiposity at early ages could act on breast carcinogenesis through other hormonal pathways (e.g.insulin-like growth factor (IGF)-I and IGF binding protein-3 [40]). The association between body size in early life and adipokine levels (e.g. leptin and adiponectin) and inflammatory marker levels (e.g. C-reactive protein (CRP)) in adulthood and their roles in breast carcinogenesis should also be explored further [41].

Although body size in early life has been consistently associated with reduced breast cancer risk, the findings on how body size in early life influences tumor characteristics are not consistent. While Baer et al. [9] found stronger inverse relationships for ER-negative tumors irrespective of the menopausal status, Bardia et al. [15] and Fagherazzi et al. [16] reported the strongest effect for ER+/PR– tumors and ER+/PR+ tumors in postmenopausal women, respectively. Although we observed a difference in the associations between body size at age 7 and ER status in KARMA, this result was not replicated in LIBRO1. In addition, our results showed that among breast cancer cases, a larger body size at age 7 was associated with smaller tumor size, although this association was not statistically significant. However, the association with tumor size was significant and was stronger for body size at age 18. A likely explanation is that body size at age 18 is inversely associated with mammographic density, which is the amount of radiographically dense tissue in the breast [42]. High mammographic density has the propensity to mask tumors on a mammogram during screening. Large body size at age 18 is highly correlated with higher BMI in adulthood, which is in turn highly correlated with less dense breasts, making it possible to detect tumors at an early stage when they are still relatively small [43]. In our data, body size at age 18 is negatively associated with percent mammographic density, after adjusting for age, BMI at questionnaire response, and menopausal status at breast cancer diagnosis (β coefficient (95% CI) in linear regression comparing large to small body size: −1.48 (−2.03 to −0.93)). Stratifying the analyses by high (≥25%) or low (<25%) percent mammographic density revealed a stronger inverse relationship between body size at age 18 and tumor size among women with low mammographic density (Table 7), suggesting that while mammographic density is likely to affect tumor size, body size at age 18 might be also associated with smaller tumor size through other mechanisms.

**Table 7:** Odds ratios (ORs) and corresponding 95% confidence intervals (CI) for the associations between body size at age 18 years and tumor size in KARMA

Body size at age 18	T0–T1 (*n*)	T2–T4 (*n*)	Multivariable OR (95% CI)^a^
*Subset of women with percent mammographic density <25%*			
Small	81	43	1.00 (Reference)
Medium	223	61	0.53 (0.33–0.85)
Large	33	6	0.34 (0.13–0.88)
*P*_trend_			0.008
*Subset of women with percent mammographic density ≥25%*			
Small	87	49	1.00 (Reference)
Medium	143	68	0.83 (0.51–1.33)
Large	15	5	0.56 (0.19–1.68)
*P*_trend_			0.249

*T* tumor size

^a^Adjusted for age, age at menarche, number of children, use of hormone replacement therapy and family history of breast cancer

Our study is unique as it was based on two independent population-based case-control studies with a large number of breast cancer cases and matched controls including both premenopausal and postmenopausal women. In addition, we used the same validated pictogram to assess early life body size, had detailed information on covariates in both studies and also examined variation by tumor subtypes. However, some limitations warrant discussion. First, we depended on women’s recall of their body size at earlier ages, which could be subject to measurement bias. However, it is unlikely that women with breast cancer reported body size in early life differently from controls. No women in the validation study reported somatotypes greater than level 7; thus the effect of extreme body fatness could not be assessed [25]. On the other hand, obese women currently tend to underestimate their body size at earlier ages than those who are not obese. This differential reporting of body size could exaggerate the risk of breast cancer among women who were small at young ages. Second, as the control population in our study was recruited among women who attended mammographic screening, there is a possibility that they might have come from higher socio-economic backgrounds. However, as all women in Sweden have essentially the same access to health care, and screening is offered as a nationwide program, the controls should be highly representative of the general population. Third, even though we adjusted for adult BMI in our analyses, many researchers argue that adult BMI should be on the causal pathway from early life body sizes to breast cancer; hence, adjustment of current BMI in the multivariable model can introduce over-adjustment bias and tend to pull the estimates towards null [44].

## Conclusion

In conclusion, our data provided further support for an inverse relationship between early life body size and breast cancer in adulthood. We also showed a significant association between body size at age 18 years and tumor size, which could potentially be mediated by mammographic density. The public health implications of this study should be interpreted cautiously and in consideration of other comorbidities in adulthood.

## Additional file

Additional file 1: Table S1. Distributions of somatotype at age 7 and 18 years and body size category change between age 7 and 18. **Table S2.** Degree of missingness for tumor characteristic information retrieved from the Swedish Cancer Registry. **Table S3.** Degree of missingness for participant characteristics (*n*, %). (DOCX 24 kb)

## Abbreviations

BMI: Body mass index
*BRCA1*: Breast cancer susceptibility gene 1
*BRCA2*: Breast cancer susceptibility gene 2
CAHRES: Cancer Hormone Replacement Epidemiology in Sweden
CI: Confidence interval
ER: Estrogen receptor
GLOBOCAN: Global Burden of Cancer Study
HRT: Hormone replacement therapy
KARMA: Karolinska mammography project for risk prediction of breast cancer
LIBRO1: Linné-bröst 1
MET: Metabolic equivalent of tasks
OR: Odds ratio
PR: Progesterone receptor
SD: Standard deviation
