# Supplementary material for: Body size in early life and risk of breast cancer
Source: Breast Cancer Res. 2017 Jul 21;19:84. doi: 10.1186/s13058-017-0875-9 (PMC5521119; doi:10.1186/s13058-017-0875-9)
Supplement: Additional file 1: Table S1. — Distributions of somatotype at age 7 and 18 years and body size category change between age 7 and 18. Table S2. Degree of missingness for tumor characteristic information retrieved from the Swedish Cancer Registry. Table S3. Degree of missingness for participant characteristics (n, %). (DOCX 24 kb) [file 13058_2017_875_MOESM1_ESM.docx]

**Table S1: Distributions of somatotype at age 7 and 18 years and body size category change between age 7 and 18**

|  | KARMA | | LIBRO1 | |
| --- | --- | --- | --- | --- |
|  | Case (*n*=2272) | Control (*n*=10468) | Case (*n*=4459) | Control (*n*=18237) |
| *Somatotype at age 7* |  |  |  |  |
| S1 | 584 (25.7) | 2449 (23.4) | 1299 (29.1) | 4244 (23.3) |
| S2 | 743 (32.7) | 3195 (30.5) | 1428 (32.0) | 5626 (30.9) |
| S3 | 471 (20.7) | 2286 (21.8) | 860 (9.3) | 3922 (21.5) |
| S4 | 302 (13.3) | 1538 (14.7) | 498 (11.2) | 2700 (14.8) |
| S5 | 123 (5.4) | 711 (6.8) | 292 (6.6) | 1259 (6.9) |
| S6 | 38 (1.7) | 247 (2.4) | 63 (1.4) | 401 (2.2) |
| S7 | 11 (0.5) | 31 (0.3) | 16 (0.4) | 69 (0.4) |
| S8 | 0 (0.0) | 9 (0.1) | 2 (0.0) | 12 (0.1) |
| S9 | 0 (0.0) | 2 (0.0) | 1 (0.0) | 4 (0.0) |
|  |  |  |  |  |
| *Somatotype at age 18* |  |  |  |  |
| S1 | 124 (5.5) | 466 (4.5) | 316 (7.1) | 836 (4.6) |
| S2 | 581 (25.6) | 2576 (24.6) | 1166 (26.2) | 4397 (24.1) |
| S3 | 884 (38.9) | 3819 (36.5) | 1689 (37.9) | 6532 (35.8) |
| S4 | 523 (23.0) | 2614 (24.9) | 949 (21.3) | 4603 (25.2) |
| S5 | 121 (5.3) | 774 (7.4) | 277 (6.2) | 1425 (7.8) |
| S6 | 32 (1.4) | 169 (1.6) | 46 (1.0) | 342 (1.9) |
| S7 | 7 (0.3) | 38 (0.4) | 10 (0.2) | 72 (0.4) |
| S8 | 0 (0.0) | 5 (0.1) | 1 (0.0) | 25 (0.1) |
| S9 | 0 (0.0) | 6 (0.1) | 0 (0.0) | 2 (0.0) |
|  |  |  |  |  |
| *Body size category change between age 7 and 18* | | | | |
| Decreased | 181 (12.0) | 898 (12.6) | 382 (12.9) | 1505 (12.2) |
| Unchanged | 1307 (86.6) | 6137 (85.8) | 2517 (85.2) | 10608 (85.9) |
| Increased | 22 (1.5) | 119 (1.7) | 55 (1.9) | 240 (1.9) |

**Table S2. Degree of missingness for tumor characteristic information retrieved from the Swedish Cancer Registry.**

|  | KARMA (*n*=2272 cases) | | LIBRO1 (*n*=4459 cases) | |
| --- | --- | --- | --- | --- |
| Tumor characteristic | Available (*n*, %) | Missing (*n*, %) | Available (*n*, %) | Missing (*n*, %) |
| ER | 1284 (56.5) | 988 (43.5) | 3463 (78.3) | 966 (21.7) |
| T | 1411 (62.1) | 861 (37.9) | 3269 (73.3) | 1190 (26.7) |
| N | 1398 (61.5) | 874 (38.5) | 3256 (73.1) | 1203 (26.9) |
| M | 1414 (62.2) | 858 (37.8) | 3261 (73.1) | 1198 (26.9) |

**Table S3. Degree of missingness for participant characteristics (*n*, %).**

|  | **KARMA** | | **LIBRO1** | |
| --- | --- | --- | --- | --- |
| **Variables** | **Cases**  **(*n*=3448)** | **Controls**  **(*n*=13505)** | **Cases**  **(*n*=5265)** | **Controls**  **(*n*=20449)** |
| **Demographics** |  |  |  |  |
| Age at questionnaire, years | 0 (0) | 0 (0) | 0 (0) | 0 (0) |
| Body size at age 7 | 383 (11.1) | 392 (2.9) | 430 (8.2) | 645 (3.2) |
| Body size at age 18 | 296 (8.6) | 73 (0.5) | 249 (4.7) | 90 (0.4) |
| BMI at questionnaire, kg/m^2^ | 293 (8.5) | 83 (<0.1) | 351 (6.7) | 0 (0) |
|  |  |  |  |  |
| **Reproductive health factors** |  |  |  |  |
| Age at menarche, years | 383 (11.1) | 422 (3.1) | 344 (6.5) | 620 (3.0) |
| Number of children | 333 (9.7) | 166 (1.2) | 224 (4.3) | 258 (1.3) |
| Menopause status | 0 (0) | 0 (0) | 0 (0) | 0 (0) |
| Ever used HRT | 365 (10.6) | 270 (2.0) | 294 (5.6) | 392 (1.9) |
| Family history of breast cancer | 462 (13.4) | 567 (4.2) | 339 (6.4) | 807 (3.9) |
